# Beyond Nutritional Treatment: Effects of Fitwalking on Physical Capacity and Intestinal Barrier Integrity in BMI-Stratified IBS Patients

**DOI:** 10.3390/nu16234181

**Published:** 2024-12-02

**Authors:** Antonella Bianco, Francesco Russo, Laura Prospero, Giuseppe Riezzo, Isabella Franco, Benedetta D’Attoma, Antonia Ignazzi, Nicola Verrelli, Claudia Beatrice Bagnato, Francesco Goscilo, Domenica Mallardi, Michele Linsalata, Caterina Bonfiglio, Pasqua Letizia Pesole, Annalisa Ferro

**Affiliations:** 1 Laboratory of Movement and Wellness, National Institute of Gastroenterology IRCCS “Saverio de Bellis”, 70013 Castellana Grotte, BA, Italy; antonella.bianco@irccsdebellis.it (A.B.); isabella.franco@irccsdebellis.it (I.F.); nicola.verrelli@irccsdebellis.it (N.V.); claudia.bagnato@irccsdebellis.it (C.B.B.); 2 Functional Gastrointestinal Disorders Research Group, National Institute of Gastroenterology IRCCS “Saverio de Bellis”, 70013 Castellana Grotte, BA, Italy; laura.prospero@irccsdebellis.it (L.P.); giuseppe.riezzo@irccsdebellis.it (G.R.); benedetta.dattoma@irccsdebellis.it (B.D.); antonia.ignazzi@irccsdebellis.it (A.I.); francesco.goscilo@irccsdebellis.it (F.G.); domenica.mallardi@irccsdebellis.it (D.M.); michele.linsalata@irccsdebellis.it (M.L.); 3 Data Science Unit, National Institute of Gastroenterology IRCCS “Saverio de Bellis”, 70013 Castellana Grotte, BA, Italy; catia.bonfiglio@irccsdebellis.it; 4 Core Facility Biobank, National Institute of Gastroenterology IRCCS “Saverio de Bellis”, 70013 Castellana Grotte, BA, Italy; letizia.pesole@irccsdebellis.it; 5 Laboratory of Clinical Pathology, National Institute of Gastroenterology IRCCS “Saverio de Bellis”, 70013 Castellana Grotte, BA, Italy; annalisa.ferro@irccsdebellis.it

**Keywords:** aerobic exercise, I-FABP, irritable bowel syndrome, obesity, walking, zonulin

## Abstract

**Background:** Irritable bowel syndrome (IBS) and obesity are associated with intestinal barrier alterations that result in low-grade inflammation. Zonulin and intestinal fatty acid-binding protein (I-FABP) assess gut barrier health, while urinary indican concentrations reflect dysbiosis in the small intestine. Physical activity, such as Fitwalking, aids weight management and improves intestinal permeability. This study assesses the impact of a 12-week Fitwalking program on intestinal barrier health in IBS patients categorized by body mass index (BMI). **Methods:** Fifty-seven mild IBS patients were categorized as obese (OB = 18), overweight (OW = 24), or normal weight (NW = 15) and assigned to a walking group. Participants walked thrice weekly at moderate intensity for 60 min per session, using the specific Fitwalking technique, supervised by staff. **Results:** No significant changes in biochemical or anthropometric variables were observed. However, Fitwalking improved the Global Physical Capacity Score (GPCS) by 46%, 48%, and 24% in the NW, OW, and OB groups. Post-intervention, serum zonulin levels notably decreased in OB individuals, suggesting reduced inflammation. OW patients unexpectedly showed increased fecal zonulin levels. OB participants experienced decreased urinary indican levels. Zonulin levels positively correlated with BMI and inversely with GPCS. **Conclusions:** Regular exercise benefits the intestinal barrier, especially in obese IBS patients. Monitoring zonulin and I-FABP may offer insights into gut barrier integrity and GI injury severity. Future studies should explore longer intervention durations, larger populations, and advanced diagnostic tools to validate findings and investigate the mechanisms behind exercise-induced changes in intestinal permeability and gut health markers.

## 1. Introduction

Irritable Bowel Syndrome (IBS) and obesity are two significant health issues that affect individuals and the economy globally. The number of people who have been diagnosed with IBS has been increasing in recent years. In Western countries, the percentage of the population affected by IBS ranges from approximately 10% to 15% [1]. Obesity is also a major public health problem worldwide, with rates more than doubling in women and nearly tripling in men. According to the World Health Organization, worldwide, more than 650 million adults suffer from obesity [2]. In Italy, the obesity rate is 18% for both men and women [3].

Both conditions are closely linked to a decline in intestinal barrier function [4]. In recent years, there has been growing interest in the crucial role of the intestinal barrier and permeability in different diseases [5]. The intestinal barrier prevents the loss of water and electrolytes while regulating the entry of antigens and microorganisms into the body [6]. Additionally, it facilitates the exchange of molecules between the host and the environment and the absorption of dietary nutrients. The altered gut barrier and subsequent translocation of bacteria or bacterial products, inducing intestinal dysbiosis, are now recognized as significant mechanisms underlying the low-grade inflammation characteristic of both IBS and obesity [7].

Different proteins, such as zonulin [8,9] and intestinal fatty acid-binding protein (I-FABP) [10], may be considered potential markers to explore the association between these conditions and intestinal barrier integrity. The first regulates tight junctions, controlling ion and substance passage to maintain barrier integrity. Elevated zonulin levels increase permeability, allowing larger molecules into the bloodstream and triggering immune responses and inflammation, notably in autoimmune and metabolic diseases [11]. Elevated zonulin levels are associated with organ fatty infiltration, especially in the liver, muscles, and myocardium, and are linked to metabolic diseases and systemic health effects [9]. The second, I-FABP, belongs to the FABP family, a group of small intracellular proteins weighing around 14 to 15 kDa widely distributed in the cytosol of various tissues. I-FABP is expressed at high levels in mucosal layer epithelial cells. Increased plasma I-FABP levels correlate with necrotizing enterocolitis and celiac disease, representing a promising marker for assessing gut wall integrity and inflammation [10].

Beyond drugs, dietary factors, lifestyle changes, and physical exercise (PE) are potential strategies against IBS and obesity. Nutritional treatment is a valid non-pharmacological approach for managing chronic conditions such as obesity and IBS; however, physical activity (PA) represents a practical alternative intervention, especially when combined with other lifestyle changes. All these non-pharmacological approaches can affect the intestinal barrier and its permeability [12,13]. Increasing evidence supports the importance of regular aerobic exercise in promoting health [14]. Specifically, regular walking benefits obese or overweight patients, as it helps burn calories essential for weight management [15]. Moreover, walking enhances cardiovascular health, metabolism, and overall fitness.

Walking is low-impact and accessible to most people, making it an excellent choice for initiating or maintaining a fitness routine. Interest in the benefits of walking has grown in recent years because it is considered safe, comfortable, and noninvasive; it has low or no cost; and, unlike other forms of exercise, it requires no special equipment or training [16]. Combined with a balanced diet, this exercise is important for achieving and maintaining a healthy weight [15].

Fitwalking is a structured, low-intensity, non-traumatic form of aerobic walking suitable for all ages and is designed to enhance physical fitness and personal well-being. This approach improves the control and management of walking, making it more effective and efficient. The technique teaches specific movements and adjustments for proper walking, enhancing precision, speed, and efficiency [17]. This walking pattern is especially suitable for individuals with obesity or other medical conditions, as it has already been successfully applied in populations with chronic diseases, such as chronic kidney disease and immune-rheumatologic disorders [17]. Due to its non-traumatic and adaptable nature, it is particularly suitable for individuals with obesity or IBS. However, research examining the specific effects of Fitwalking on intestinal barrier integrity in IBS patients remains limited.

Our group has thoroughly documented the benefits of nutrition in improving gastrointestinal (GI) symptom profiles and psychological well-being in individuals with IBS [18,19]. Recently, our research has also focused on the impact of PA in alleviating GI symptoms [20] and enhancing physical capacity (PC), as assessed by the Global Physical Capacity Score (GPCS) in these patients [21].

GPCS is considered an index of cardiorespiratory capacity, muscle strength, and flexibility obtained by calculating the sum of three validated field tests. Our previous study correlated higher PC levels with more significant health benefits. Thus, GPCS monitoring could provide insight into the link between PA and IBS symptom severity and help healthcare professionals develop personalized treatment plans [21].

In this framework, the present study aimed to investigate the effects of a 12-week Fitwalking program, monitored through GPCS, on intestinal barrier integrity in IBS patients classified by body mass index (BMI) as normal weight (NW), overweight (OW), or obese (OB). The authors assessed changes in serum and fecal zonulin, circulating I-FABP levels, and urinary indican as markers of gut barrier function and fermentative dysbiosis [22]. By integrating PC measures with biochemical markers, this study sought to provide a comprehensive understanding of how aerobic exercise affects gut health in IBS, addressing the gap in the literature on the role of structured PA in managing IBS and obesity.

## 2. Materials and Methods

### 2.1. Study Design and Participants

This study is based on data from the clinical trial NCT05453084, registered at https://clinicaltrials.gov/ (accessed on 16 November 2023). The Local Ethics Committee approved it, and the study was carried out under the Declaration of Helsinki (Prot. No. 167/CE De Bellis). All the participants signed informed consent. The trial’s design has been previously detailed elsewhere [21]. In summary, the study involved patients suffering from symptoms resembling IBS according to Rome criteria [23]. They were either referred by local general practitioners or attended the outpatient clinic for celiac disease and functional disorders. The invitation to participate in the study was also published through online press releases, media, and correspondence with primary care physicians.

**Figure 1:**
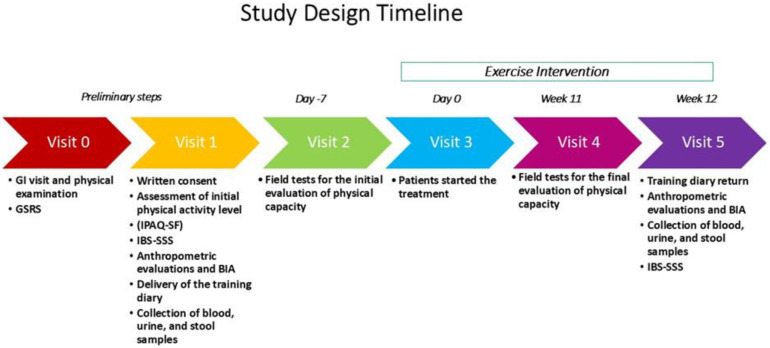
The design of the study. GI = Gastrointestinal; GSRS = Gastrointestinal Symptom Rating Scale; IPAQ-SF = International Physical Activity Questionnaire Short Form; IBS-SSS = Irritable Bowel Syndrome Severity Scoring System; BIA = Bioelectrical Impedance Analysis.

The study sample consisted of patients aged 18 to 65. The inclusion criteria were a willingness to join the walking group and a medical certificate of non-competitive sports fitness. The exclusion criteria were the existence of severe neurological, psychiatric, cardiac, metabolic, or liver diseases, GI organic disorders, patients using antidepressants, significant orthopedic or neuromuscular limitations, and absolute contraindications to exercise. The study design timeline is reported in Figure 1.

### 2.2. Data Collection

Enrolled participants completed a structured anamnestic questionnaire and two validated GI questionnaires: the Gastrointestinal Symptom Rating Scale (GSRS) [24], which uses a 7-point Likert scale to assess symptom intensity and frequency over the past seven days, and the IBS Symptom Severity Score (IBS-SSS) [25], which measures the severity of IBS symptoms (including pain, bloating, bowel habits, and impact on daily life), with a total score ranging from 0 to 500. This study specifically involved patients who complained of mild IBS symptoms (IBS-SSS total score between 75 and 175) [25].

The International Physical Activity Questionnaire, Short Form (IPAQ-SF) [26] collected information on PA levels.

The study examined the participants’ physical characteristics through various anthropometric measurements, including height, weight, waist, hip and mid–upper arm circumferences, and BMI. The BMI was calculated using accurate weight and height measurements with a SECA 700 mechanical column scale and a SECA 220 altimeter (INTERMED S.r.l., Milan, Italy). Standard laboratory techniques were used to measure biochemical features before and after the intervention. A strict protocol was followed to ensure consistency for those undergoing bioelectrical impedance analysis (BIA). This included a minimum 4-h fast, and abstaining from both alcohol and vigorous exercise for 12 h before the study.

The BIA procedure involved administering a continuous sinusoidal current (800 A) with a frequency of 50 kHz. All measurements were performed using the BIA 101 BIVA PRO device (Akern SRL, Pontassieve, Italy), which complies with the standards set forth by the European Society for Parenteral and Enteral Nutrition [27].

The BIA method evaluated parameters such as reactance (Xc) and resistance (Rz). The same device was used to measure the body cell mass, fat mass, fat-free mass, total body water, and extracellular water. Specialized software (Bodygram PLUS Software v. 1.0, Akern SRL, Pontassieve, Italy) was used to calculate these parameters based on the determined Rz and Xc values. Another important metric in the assessment was the phase angle, which was calculated as the arctangent of the Xc/Rz ratio.

Blood samples were collected between 8:00 and 9:00 a.m. following an overnight fast. Standard laboratory procedures assessed the biochemical characteristics before and after the exercise intervention. The fluorescence flow cytometry for blood cell count was conducted using the Sysmex XT-1000 automatic hematology analyzer (Dasit, Cornaredo, Milan, Italy). Fasting blood glucose, insulin, triglycerides, total cholesterol, Low-Density Lipoprotein cholesterol (LDL-cholesterol), High-Density Lipoprotein cholesterol (HDL-cholesterol), Aspartate Transaminase (AST), Alanine Transaminase (ALT), Gamma Glutamyl Transpeptidase (GGT), 25-OH Vitamin D, and high-sensitivity C-reactive protein (hs-CRP) concentrations were measured using the COBAS 8000 autoanalyzer (ROCHE Diagnostic SPA, Monza, Italy).

### 2.3. Exercise Protocol

#### 2.3.1. Physical Capacity Evaluation Test

Three validated field tests were selected to comprehensively assess the participants’ PC. These tests assessed cardiorespiratory fitness, muscular strength, and flexibility—critical components of overall PC. To ensure consistency and reliability, the tests were administered at baseline and following the 12-week intervention, adhering to standardized procedures.

The “2 km Walk Test” was employed to measure cardiorespiratory fitness, providing an indirect estimate of maximal oxygen uptake (VO_2_max). Participants walked a 2 km distance on a flat, marked outdoor course at their fastest sustainable pace. Heart rate was measured immediately upon completion using a heart rate monitor, and the time taken to complete the walk was recorded. Based on these parameters, a fitness index was calculated. This practical and non-invasive test suits diverse populations, including IBS patients classified by BMI. As a reliable indicator of cardiorespiratory health, the test reflects overall fitness and metabolic efficiency [28].

The “Hand Grip Test” assessed upper body strength, a critical component of musculoskeletal health. The participants used a calibrated hand dynamometer to determine their maximum grip strength. The test is repeated three times per hand, alternating hands with a pause between each test (30–60 s) to avoid fatigue. The average for each hand was used for analysis. Grip strength is a validated measure of overall muscle strength and is predictive of health outcomes, including functional independence and quality of life. It serves as an indirect marker of muscular endurance and strength, particularly relevant for evaluating the effects of PA interventions [29].

The “Sit and Reach Test” evaluated flexibility, specifically targeting the lower back and hamstring muscles. Participants sat on the floor, legs extended straight ahead and feet flat against a standardized sit-and-reach box. They were instructed to reach forward as far as possible without bending their knees, and the distance reached beyond their toes (measured in centimeters) was recorded. Participants performed two attempts, and the highest score was considered. Flexibility, a crucial aspect of physical fitness, is especially important in populations at risk for musculoskeletal issues. This test indicates the participants’ range of motion and functional flexibility [30].

Several standardization measures were implemented to ensure the reliability and reproducibility of the test results. All tests were conducted under the same conditions, including location, time of day, operators, and equipment. Detailed instructions and demonstrations were provided before the tests, and practice trials were allowed when necessary. Participants were encouraged to wear comfortable clothing and appropriate footwear. Furthermore, to reduce result variability, the same operators conducted the tests both before and after the intervention. This comprehensive evaluation framework allowed for a multidimensional assessment of PC, ensuring accurate and reliable data collection to measure the outcomes of the Fitwalking intervention.

#### 2.3.2. Fitwalking Intervention

Fitwalking, an aerobic activity of moderate intensity, and Walking Groups were used as PE intervention tools. The specific characteristics of the PE intervention were as follows: (a) frequency: the walks were conducted outside on an urban route three times a week, on non-consecutive days, for three months; (b) intensity: the walking exercise was of moderate intensity (60/75% of maximum heart rate); it was monitored through the heart rate monitor and personalized through Tanaka’s formula [31]; (c) type: aerobic exercise consists of walking, at a speed of 5 to 10 km/h, using the Fitwalking technique [17]; (d) time: each walk lasted 60′, for a total of 180′ per week.

Professionals with a graduate degree in preventive and adapted PA science and techniques oversaw PE. They also performed the role of the Fitwalking leader during the walks. The adherence to the exercise program was recorded.

#### 2.3.3. Training Diary

Each participant completed a daily diary documenting the type and duration of PA undertaken. After each day, participants recorded the total number of daily steps tracked by their heart rate monitor. The diary was a tool to encourage patients to engage in PA, compare participation in the walking group, and understand and quantify additional PA beyond the prescribed regimen. Upon receiving the training diary, participants were provided with comprehensive instructions on how to complete it accurately.

#### 2.3.4. Global Physical Capacity Score

PC was assessed through a series of motor tests with varying difficulty levels, validated in adult subjects to evaluate cardiorespiratory capacity, strength, and flexibility. A PC score was then computed based on the outcomes of each test. Each physical test received a score ranging from 0 to 2 using performance categories (e.g., performance above average = 2 points, average = 1 point, below average or unable to complete the test = 0 points). Subsequently, the scores from the three tests were summed to derive an overall physical ability score, with a potential score range of 0 to 6 points. The GPCS utilized in this study was adapted from the methodology proposed by Bouchard et al. [32]. One advantage of computing and using the GPCS is that it provides a comprehensive measure of physical performance, considering the different tasks involved in daily activities rather than evaluating each test individually.

### 2.4. Biomarkers of Intestinal Barrier Function and Dysbiosis

Biochemical assays were conducted at the beginning and end of the exercise intervention. Patient serum and stool samples were collected and frozen at −80 °C within 12 h. Enzyme-linked immunoassay (ELISA) kits from Immunodiagnostic AG (Bensheim, Germany) were used to detect zonulin levels in serum and fecal samples, following the manufacturer’s instructions. Normal values were below 48 ng/mL for serum and 107 ng/mL for fecal samples. I-FABP serum concentrations were measured using ELISA kits from Cloud-Clone Corp. (Houston, TX, USA). The indican levels in the urine were measured using an established colorimetric assay kit (Indican assay kit, ABNOVA Corporation, Taipei, Taiwan). According to the literature, indican levels exceeding 20 mg/L may indicate fermentative dysbiosis [22].

### 2.5. Statistical Analysis

Continuous data were reported as mean ± SEM. Categorical data are represented as numbers and percentages. IPAQ-SF was categorized according to the metabolic equivalent of task (MET) into inactive (<700 MET), sufficiently active (700–2519 MET), and active/very active (>2520 MET). The Kruskal Wallis test with Dunn’s multiple comparison tests was applied to verify group differences. Data within the groups were analyzed using the Wilcoxon rank sum test (pre- and post-walking activity). Categorical data were analyzed with the Chi-Square test. The Spearman test was performed for correlation analysis on a dataset comprising 57 XY pairs.

Multiple linear regression analysis was conducted with GPCS as the dependent variable, incorporating clinical and biochemical parameters that demonstrated significant correlations with GPCS as independent variables in a step-wise regression approach. The explained variance (adjusted R-squared) for the model was determined and assessed using an F-test. To evaluate whether each included the variable’s contribution (the regression coefficient) significantly deviated from zero, t-values and corresponding significance levels were calculated. Statistical significance was set at *p* < 0.05.

## 3. Results

### 3.1. Patients’ Characteristics

**Figure 2:**
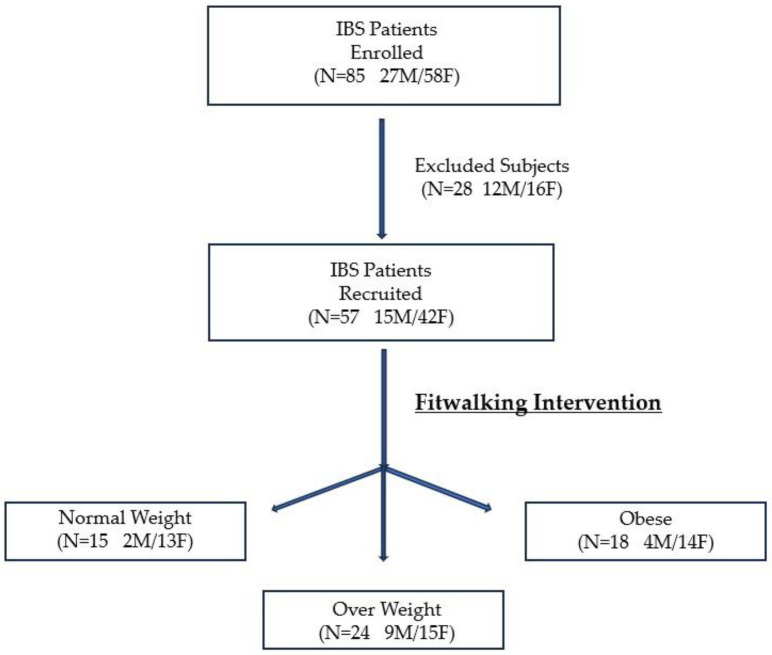
The study’s flowchart. M = Male; F = Female; IBS = Irritable Bowel Syndrome.

In the present study, 85 patients with a long-standing history of IBS but with mild IBS-SSS total score at the time of recruitment (IBS-SSS between 75 and 175) were considered. Fifty-seven IBS patients (74% women) completed the study, and 28 were excluded for various reasons (eight patients declined to participate because they had family obligations, 16 because they had work commitments, three because they needed antibiotics or probiotics for acute infections, and one because he had undergone major surgery). All participants followed the same exercise program. The study flow is illustrated in Figure 2.

Table 1 shows the patients’ anthropometric characteristics. At the start of the program, significant differences were observed among the three groups across all parameters. As expected, clear differences were found between OB and NW patients and between OB and OW patients. A description of the anthropometric and bioimpedance characteristics of IBS patients stratified by BMI and sex at baseline (Pre) and after (Post) the exercise intervention is reported in Table S1.

No significant differences were found between the groups regarding IPAQ-SF. After the exercise intervention, the anthropometric parameters remained significantly different among the three groups. The Fitwalking program did not induce any changes, except for a significant reduction in the waist circumference in OW patients (*p* = 0.0057).

### 3.2. Effects of Fitwalking on GPCS

At the start of the study, the NW group already had higher levels of GPCS (3.48 ± 0.38) compared to both OW (2.37 ± 0.29) and OB patients (2.28 ± 0.36). However, no statistically significant differences were found.

**Figure 3:**
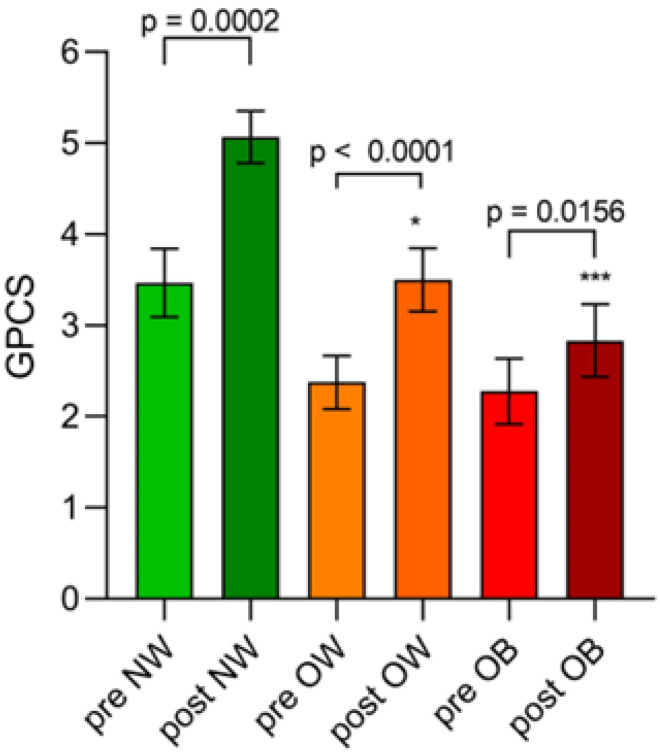
The effects of walking on the Global Physical Capacity Score (GPCS). NW: Normal Weight; OW: Overweight; OB: Obese. Data are expressed as mean ± SEM. Data within the groups were analyzed by the Wilcoxon rank sum test (pre- and post-exercise intervention). The Kruskal Wallis test was applied to verify group differences (Dunn’s multiple comparisons test * *p* = 0.0127 between NW group and OW group. *** *p* = 0.0005 between NW group and OB group).

At the end of the treatment, all groups benefited from the walking exercise, experiencing a significant increase in GPCS by 46%, 48%, and 24%, respectively. However, mean GPCS values of the NW group (5.07 ± 0.78) were significantly higher than those of OW (3.5 ± 0.35) and OB patients (2.83 ± 0.39) as revealed by the Kruskal Wallis test with Dunn’s multiple comparison tests (*p* = 0.0127 between NW and OW; *p* = 0.0005 between NW and OB) (Figure 3).

The impact of the Fitwalking program on GPCS was evident, as indicated by the significant increase in the values of all three groups at the end of the 12-week treatment (Wilcoxon test: *p* = 0.0002 in NW, *p* < 0.0001 in OW, and *p* = 0.0156 in OB).

### 3.3. Effects of Fitwalking on the Biochemical Parameters

Biochemical analyses were conducted at the beginning and end of the exercise intervention, as shown in Table 2. Before the intervention, insulin concentrations were significantly higher in OW patients than in NW patients (*p* = 0.0002), although they were within the normal range for both groups. Similarly, OW patients had slightly higher ALT levels than NW patients (*p* = 0.02), but OB patients did not.

Regarding lipid profile, HDL cholesterol levels were lower in OW and OB patients. OB subjects showed significantly reduced HDL-cholesterol levels (*p* = 0.03) and significantly elevated triglyceride levels (*p* = 0.008) compared to NW patients. All groups had total cholesterol levels above 200 mg/dL before and after treatment.

At the end of the study, insulin levels remained higher in OB patients than in the NW group (*p* = 0.0001). Moreover, ALT levels remained higher in the OW group than in the NW group (*p* = 0.03). Additionally, HDL cholesterol levels were lower in OB patients than in the NW group (*p* = 0.009). The walking exercise program did not cause any changes in the biochemical parameters within the three groups.

### 3.4. Effects of Fitwalking on the Markers of Intestinal Barrier and Fermentative Dysbiosis

Table 3 reports the effects of walking on markers of intestinal barrier integrity and function. Before the walking exercise, only the NW patients’ group had serum zonulin concentrations below the critical value. There was a significant difference in serum zonulin between the NW and OB groups (*p* = 0.0102). After the treatment, serum zonulin levels decreased in all three groups with no significant difference. The Fitwalking program significantly reduced serum zonulin levels in the OB group (* *p* = 0.0268). However, fecal zonulin concentrations did not differ between the groups before and after treatment. Nonetheless, it increased due to the walking exercise, reaching a statistically significant increase in the OW subjects (** *p* = 0.0164).

There were no significant differences in the serum I-FABP levels among the three groups before and after treatment. All three groups showed urinary indican levels higher than the specific cut-off before and after the Fitwalking program. OW and OB subjects did not have significantly higher indican levels than NW patients. The Fitwalking program significantly decreased indican levels only in the OB group (*p* = 0.0304).

### 3.5. Correlations Between BMI, GPCS, and the Markers of Intestinal Barrier Function and Integrity

At the study’s beginning, there was a significant negative correlation between BMI and GPCS (r = −0.31, 95% CI: −0.5290 to −0.03953, *p* = 0.021). This correlation remained stronger following the intervention (r = −0.5046, 95% CI: −0.6805 to −0.2737, *p* < 0.0001).

**Figure 4:**
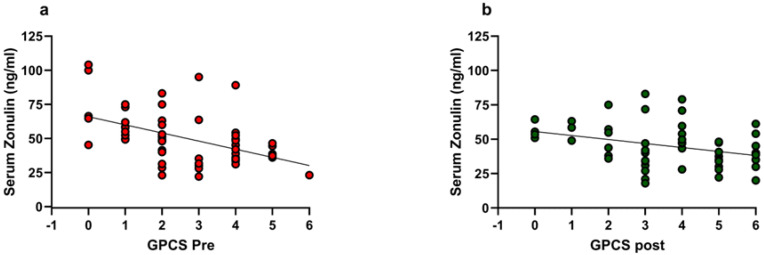
Correlations between serum zonulin and GPCS pre- and post-exercise intervention. GPCS: Global Physical Capacity Score. The Spearman correlation analysis was performed on the whole dataset. Panel (**a**): Pre-exercise intervention (r = −0.5167, 95%CI = −0.6892 to −0.2888, *p* < 0.0001). Panel (**b**): Post-exercise intervention (r = −0.3580, 95%CI = −0.5711 to −09961, *p* = 0.0063).

At baseline, BMI positively correlated with serum zonulin levels (r = 0.36, 95% CI: 0.09933 to 0.5709, *p* = 0.0063). Serum zonulin and GPCS also showed a strong negative correlation (r = −0.5167, 95% CI: −0.6892 to −0.2888, *p* < 0.0001) (Figure 4, Panel a). Post-intervention, this negative association persisted but was weaker (r = −0.3580, 95% CI: −0.5711 to −0.09961, *p* = 0.0063) (Figure 4, Panel b).

No significant correlations were detected between fecal zonulin or serum I-FABP levels with BMI or GPCS at any time.

Multiple linear regression analysis revealed that baseline GPCS levels were significantly predicted by BMI and serum zonulin (F = 11.015, df = 2, *p* < 0.001, adjusted R^2^ = 0.263). Specifically, higher BMI (β = −0.067, *p* = 0.043) and elevated serum zonulin (β = −0.033, *p* = 0.001) were both associated with lower GPCS (Table 4). These results suggest that clinical and biochemical variables, particularly BMI and serum zonulin, independently influence PC at baseline.

## 4. Discussion

IBS and obesity are closely related to impaired intestinal barrier functions, and zonulin and I-FABP proteins may serve as reliable markers of gut health and inflammation. Exercise and diet can also impact the intestinal barrier, with PA being crucial in addressing obesity-related issues.

Our intervention, which involved 12 weeks of Fitwalking, led to significant improvements in PC among all groups of IBS patients, classified according to their BMI. This study focused on patients experiencing mild IBS symptoms, aiming to create a homogeneous group within a specific IBS-SSS range to minimize the impact of symptom variability on the primary outcome measures, especially those related to intestinal barrier integrity. Since more pronounced intestinal barrier changes in IBS are often associated with severe symptoms and heightened sensitivity to somatic and visceral stimuli [33], selecting patients with moderate symptom severity helped control for potential confounding effects from more severe IBS profiles. Consequently, no significant changes in biochemical parameters were observed due to Fitwalking.

Serum zonulin levels decreased in all groups after exercise intervention, particularly in OB patients. On the other hand, fecal zonulin concentrations increased in OW subjects. Urinary indican levels were elevated in all three groups before the study but decreased significantly after the Fitwalking intervention, only in the OB group, remaining above the cut-off level.

The literature highlights numerous benefits of PA for individuals with obesity and related conditions [14,34], promoting diversity in intestinal microbiota and overall gut health [35]. Studies suggest aerobic exercise is better than resistance training in enhancing fitness, reducing body fat, and regulating blood pressure [36]. Its appeal lies in its accessibility, minimal skill requirements, and low risk of injury. Adaptable to various speeds and intensities, walking allows patients to participate individually or in groups without specialized equipment [15], making it an enjoyable option for OW, OB, or untrained individuals. Fitwalking has been used as a specific model of structured exercise. It improves walking control, making it more effective and efficient. It teaches specific movements and adjustments that increase the incisiveness, speed, and economy of movement [17]. Regarding the available tools for monitoring exercise, the GPCS provides a comprehensive measure of PC [21]. As indicated by the multiple regression model, the basal GPCS, serving as a comprehensive indicator of physical health in our IBS patients at the start of the study, was affected by both a clinical variable, BMI, and a biochemical marker, serum zonulin, in an inverse relationship. Zonulin acts as an indirect marker of IP disruption, which, as reported in the literature, can lead to localized and systemic GI and extra-GI conditions [37]. Beyond the established link between impaired IPs and metabolic diseases—including obesity—recent research highlights the protective role of aerobic exercise in maintaining intestinal barrier integrity [38]. PE may reduce zonulin levels, enhance mucin production, and decrease intestinal inflammation, improving the intestinal barrier health [39]. At the end of treatment, the GPCS score improved, confirming the effectiveness of the walking program in improving fitness, as reported by other authors [14,40].

The proposed walking program did not aim to reduce weight or improve biochemical parameters. The anthropometric and BIA parameters remained significantly different among the three groups during recruitment and after the Fitwalking program. However, the waist circumference of the OW group showed a significant reduction.

All biochemical parameters except total cholesterol levels were within normal ranges in the three groups at baseline. Significant differences were observed in hematochemical parameters among the groups. Initially, OB subjects exhibited higher insulin and triglyceride levels and lower HDL levels than NW subjects, while OW patients had higher ALT levels. Despite an overall improvement at the end of the project, group disparities persisted. Interestingly, OB patients showed no abnormal values apart from cholesterol levels, possibly indicating metabolically healthy obesity (MHO). MHO is characterized by normal glucose and lipid metabolism, no hypertension, and better cardiorespiratory fitness than metabolically unhealthy obese individuals [41].

Fitwalking seems to enhance the integrity of the intestinal barrier effectively. Recent research indicates that prolonged exercise can improve intestinal permeability considerably, particularly among patients with type 2 diabetes [42]. Moderate-intensity exercise has also significantly enhanced the expression of various intestinal tight junction proteins and zonulin [42]. The latter is a serum marker for intestinal barrier health [43], and our findings indicate significantly higher baseline serum concentrations of this protein in OB subjects than in NW and OW subjects. Circulating zonulin levels correlated positively with BMI, while both factors correlated negatively with GPCS, highlighting the close relationship between impaired intestinal barrier integrity and increased body weight as well as how both conditions worsen in situations of reduced PC. In line with the above finding, serum zonulin levels decreased in all groups after the exercise; however, a statistically significant reduction was observed only in OB subjects.

Previous studies linked high serum zonulin to obesity and increased energy intake [44]. Furthermore, elevated zonulin levels correlate with greater waist circumference, diastolic blood pressure, glucose levels, and hyperlipidemia rather than GI symptoms [45].

Zonulin reflects shifts in intestinal permeability due to bacterial translocation, immune activation, and low-grade inflammation [46,47,48]. More interestingly, its production in extra-intestinal tissues like abdominal adipose tissue and liver [49,50,51], suggests that elevated levels could indicate heightened intestinal permeability [52] and may be present in elevated concentrations in conditions like obesity, metabolic syndrome, and inflammation. Consequently, serum levels tend to rise in OW/OB individuals.

Assessing serum zonulin levels may provide a broader perspective on inflammation across various organs and systems, including metabolic, cardiovascular, and joint-related, rather than solely giving information on the intestinal tract. This condition is affected by shifts in immunity, compromised intestinal barrier function, and intestinal flora dysbiosis. Our hypothesis aligns with the discovery that only the NW group initially exhibited serum zonulin levels below the cutoff. In contrast, the OW and OB groups had higher values, indicative of inflammation.

Fecal zonulin levels exceeded the cut-off in OW and OB patients, too. Unexpectedly, walking exercise increased fecal levels in the OW group, unlike the NW and OB groups, where no significant changes occurred. Exercise-induced intestinal hyper-permeability can occur in healthy individuals who engage in vigorous aerobic exercise, such as athletes, rather than untrained individuals [53]. However, the underlying reasons are not fully understood due to the lack of data. We plan to perform additional studies with a larger sample size for deeper insights.

Regarding I-FABP, this tiny 15 kDa protein, mainly expressed by enterocytes, reflects epithelial damage. No significant differences in I-FABP levels were found among the three groups, pre- and post-treatment. Previous studies have shown that plasma I-FABP increases post-exercise, especially during strenuous activity, causing intestinal stress from factors like high core body temperature, oxidative stress, mechanical strain (e.g., running), and reduced splanchnic blood flow due to redistribution towards active muscles and organs [54]. While our study did not show reduced serum I-FABP levels, it did not indicate elevation, suggesting our exercise intensity was likely appropriate, though intervention duration may have been insufficient.

The divergent responses of serum zonulin and I-FABP may stem from their distinct reactions to environmental stimuli. Monitoring I-FABP levels can aid in the early detection of the severity of GI injuries. Elevated zonulin levels signal heightened intestinal permeability during exercise. However, unlike I-FABP, zonulin cannot be an early biomarker to estimate the extent of GI injury [55].

A compromised gut barrier often accompanies disturbances in intestinal microbiota [42,43]. Urinary indican, an indirect marker of intestinal dysbiosis, stems from bacterial action on tryptophan, with levels above 20 mg/L indicating small intestinal dysbiosis [19]. In our study, all groups exhibited dysbiosis, with OB individuals showing the highest levels. While Fitwalking reduced indican levels in OB subjects, they remained above the cut-off. Even the NW group displayed elevated levels, possibly due to mild IBS symptoms affecting bacterial flora [56].

The present study has positive results but also some limitations that should be considered. Among its strengths, it addresses the impact of Fitwalking on physical condition and intestinal barrier integrity in overweight and obese patients with IBS, representing a novel and valuable contribution to the field. The intervention was meticulously supervised by trained professionals, who provided a personalized approach tailored to each participant, ensuring full adherence to the program. The training protocol was also developed in accordance with internationally recognized guidelines, which improved its validity and optimized its applicability for IBS patients.

However, certain limitations should be acknowledged. The sample’s larger percentage of female participants may have introduced a potential sex-related bias and limited the findings’ generalizability. Still, it also reflects the higher prevalence of IBS among women. Additionally, the 12-week duration of the intervention may have been insufficient to detect significant changes in body weight, a variable that typically requires longer-term observation. Finally, while informative, the exclusive reliance on circulating markers to evaluate intestinal barrier integrity may lack the depth provided by more comprehensive assessments such as lactulose/mannitol permeability testing or 16S rRNA sequencing to evaluate changes in the gut microbiota.

## 5. Conclusions

This study highlights the value of structured aerobic exercise, such as Fitwalking, in improving intestinal barrier integrity and overall well-being in IBS patients. The observed improvements in PC (GPCS) and reductions in serum zonulin levels, particularly in obese individuals, suggest a unique relationship between exercise and gut health that is independent of weight loss or dietary changes. These findings provide valuable insights into non-pharmacological approaches for managing IBS and obesity.

A key strength of this study is its focus on IBS patients with mild symptoms, which reduces variability and clarifies the biochemical and physical outcomes of Fitwalking. By examining relationships among BMI, GPCS, and serum zonulin, this research demonstrates the systemic benefits of moderate-intensity exercise in enhancing intestinal permeability, particularly in overweight and obese populations.

The novelty lies in using Fitwalking as a practical and enjoyable aerobic intervention for IBS patients. Unlike studies on dietary changes, this research shows that moderate exercise alone can positively influence gut health markers, including serum zonulin and urinary indican. These findings offer a model for integrating exercise into IBS and obesity management programs, advancing understanding of PA’s role in gut health beyond weight loss.

Persistent elevations in fecal zonulin and urinary indican in some subgroups highlight the need for further research into the mechanisms underlying exercise’s impact on gut health. Future studies should explore longer interventions, larger populations, and advanced diagnostic tools like permeability tests and microbiome sequencing to validate and expand these findings.

In conclusion, this study positions moderate-intensity exercise, particularly Fitwalking, as an impactful strategy for managing gut health. It underscores the interconnection of physical fitness and GI health, offering a new perspective on clinical practice and public health.

## Figures and Tables

**Table 1:** Characteristics of IBS patients according to BMI at the baseline (Pre) and after (Post) exercise intervention.

		NW (n. 15)	OW (n. 24)	OB (n. 18)	*p*-Value
Sex (Male/Female)		2 M/13 F	9 M/15 F	4 M/14 F	
Age (years)		55.07 ± 1.29 ^a^	56.33 ± 1.23 ^a^	53.78 ± 2.35 ^a^	0.85
Waist Circumference	Pre	78.47 ± 1.68 ^a^	91.41 ± 1.50 ^b^*	107.3 ± 2.23 ^c^	<0.0001
	Post	77.66 ± 1.72 ^a^	89.81 ± 1.57 ^b^*	106 ± 2.32 ^c^	<0.0001
Hip Circumference	Pre	97.43 ± 1.29 ^a^	103 ± 1.27 ^a^	116 ± 2.75 ^b^	<0.0001
	Post	97.31 ± 1.39 ^a^	101.9 ± 1.20 ^a^	114.5 ± 2.69 ^b^	<0.0001
Waist/Hip Ratio	Pre	0.80 ± 0.02 ^a^	0.88 ± 0.02 ^ab^	0.87 ± 0.06 ^b^	0.02
	Post	0.81 ± 0.03 ^a^	0.87 ± 0.02 ^ab^	0.93 ± 0.02 ^b^	0.003
PhA (degrees)	Pre	5.94 ± 0.26 ^a^	6.41 ± 0.19 ^ab^	6.71 ± 0.15 ^b^	0.033
	Post	6.37 ± 0.24 ^a^	6.34 ± 0.16 ^a^	6.64 ± 0.20 ^a^	0.56
BCM (kg)	Pre	24.4 ± 1.37 ^a^	28.48 ± 1.33 ^ab^	31.61 ± 1.41 ^b^	0.001
	Post	25.13 ± 1.36 ^a^	28.18 ± 1.23 ^ab^	31.24 ± 1.24 ^b^	0.001
FM (kg)	Pre	19.95 ± 2.86 ^a^	23.18 ± 1.17 ^a^	36.61 ± 2.51 ^b^	<0.001
	Post	16.25 ± 0.82 ^a^	22.73 ± 1.01 ^b^	37.98 ± 2.67 ^c^	<0.001
FFM (kg)	Pre	45.71 ± 1.73 ^a^	51.10 ± 1.93 ^ab^	55.32 ± 2.04 ^b^	0.003
	Post	45.20 ± 1.59 ^a^	50.81 ± 1.81 ^ab^	55.26 ± 1.91 ^b^	0.0004
TBW (liters)	Pre	33.47 ± 1.32 ^a^	37.11 ± 1.44 ^ab^	40.47 ± 1.49 ^b^	0.004
	Post	32.83 ± 1.13 ^a^	37.06 ± 1.34 ^ab^	40.40 ± 1.42 ^b^	0.0005
ECW (liters)	Pre	15.43 ± 0.60 ^a^	16.30 ± 0.60 ^a^	17.27 ± 0.57 ^a^	0.080
	Post	14.46 ± 0.38 ^a^	16.38 ± 0.54 ^ab^	17.45 ± 0.68 ^b^	0.004
IBS-SSS Total Score	Pre	94.33 ± 5.50 ^a^	94.38 ± 4.15 ^a^	92.22 ± 4.79 ^a^	0.99
	Post	91.67 ± 6.95 ^a^	92.08 ± 4.39 ^a^	89.72 ± 4.47 ^a^	0.96
IPAQ-SF Categories °					
Inactive		4 (26.7%)	7 (29.2%)	4 (22.2%)	
Sufficiently Active		8 (53.3%)	11 (45.8%)	12 (66.7%)	0.730
Active/Very Active		3 (20.0%)	6 (25%)	2 (11.1%)	

IBS: Irritable Bowel Syndrome; BMI: Body Mass Index; NW: Normal Weight; OW: Overweight; OB: Obese; PhA: phase angle; BCM: body cell mass; FM: fat mass; FFM: fat-free mass; TBW: total body water; ECW: extracellular water. IBS-SSS: Irritable Bowel Syndrome Severity Scoring System. IPAQ-SF: International Physical Activity Questionnaire-Short Form Continuous data reported as Mean ± SEM. Categorical data are represented as numbers and percentages. Data within the groups were analyzed by the Wilcoxon rank sum test (pre- and post-exercise intervention); * Statistical significance *p* < 0.05. The Kruskal Wallis test with Dunn’s multiple comparison tests was applied to verify differences among groups pre- and post-exercise intervention; different letters differ significantly. ° Categorical data were analyzed with the Chi-Square test; statistical significance *p* < 0.05.

**Table 2:** Biochemical characteristics of the participants categorized according to BMI at the baseline (Pre) and after (Post) exercise intervention.

		NW (n. 15)	OW (n. 24)	OB (n. 18)	*p*-Value
TSH (μU/mL)	Pre	2.83 ± 0.52 ^a^	4.75 ± 0.93 ^a^	5.59 ± 1.28 ^a^	0.62
	Post	2.33 ± 0.30 ^a^	4.46 ± 0.92 ^a^	6.27 ± 1.38 ^a^	0.19
fT3 (pg/mL)	Pre	3.19 ± 0.11 ^a^	3.30 ± 0.07 ^a^	3.31 ± 0.09 ^a^	0.69
	Post	3.24 ± 0.10 ^a^	3.30 ± 0.09 ^a^	3.39 ± 0.10 ^a^	0.58
fT4 (pg/mL)	Pre	1.12 ± 0.06 ^a^	1.01 ± 0.04 ^a^	1.03 ± 0.04 ^a^	0.40
	Post	1.06 ± 0.09 ^a^	1.06 ± 0.05 ^a^	1.07 ± 0.05 ^a^	0.88
Glucose (mg/dL)	Pre	87.53 ± 2.88 ^a^	92.79 ± 2.62 ^a^	102.8 ± 9.88 ^a^	0.22
	Post	88.87 ± 2.71 ^a^	90.79 ± 2.38 ^a^	94.33 ± 3.44 ^a^	0.44
Insulin (μU/mL)	Pre	5.92 ± 0.49 ^a^	8.55 ± 0.69 ^ab^	13.21 ± 1.54 ^b^	0.0002
	Post	6.17 ± 0.79 ^a^	9.18 ± 1.07 ^a^	13.71 ± 1.39 ^b^	0.0001
Uric acid (mg/dL)	Pre	36.60 ± 2.44 ^a^	37.46 ± 1.23 ^a^	39.11 ± 2.39 ^a^	0.76
	Post	36.20 ± 2.56 ^a^	37.67 ± 2.30 ^a^	37.78 ± 1.91 ^a^	0.89
25-OH-Vitamin D (ng/mL)	Pre	30.93 ± 3.62 ^a^	24.93 ± 2.11 ^a^	22.62 ± 1.72 ^a^	0.23
	Post	34.51 ± 3.19 ^a^	29.19 ± 1.84 ^a^	25.51 ± 1.60 ^a^	0.15
γGT (U/L)	Pre	28.80 ± 9.35 ^a^	20.88 ± 2.44 ^a^	21.67 ± 2.81 ^a^	0.55
	Post	23.87 ± 7.53 ^a^	30.67 ± 8.61 ^a^	22.83 ± 3.45 ^a^	0.36
ALT (U/L)	Pre	18.07 ± 0.89 ^a^	27.25 ± 3.16^b^	25.28 ± 2.20 ^ab^	0.02
	Post	17.40 ± 1.46 ^a^	26.79 ± 3.42 ^ab^	23.72 ± 1.64 ^b^	0.03
AST (U/L)	Pre	19.27 ± 1.73 ^a^	23.54 ± 1.29 ^a^	20.67 ± 1.39 ^a^	0.06
	Post	19.53 ± 1.49 ^a^	22.46 ± 2.18 ^a^	19.06 ± 0.99 ^a^	0.56
Total Cholesterol (mg/dL)	Pre	203.3 ± 9.73 ^a^	205.5 ± 5.58 ^a^	210.7 ± 8.00 ^a^	0.60
	Post	205.7 ± 10.57 ^a^	207.2 ± 6.68 ^a^	217.2 ± 9.13 ^a^	0.47
HDL Cholesterol (mg/dL)	Pre	69.05 ± 5.61 ^a^	55.21 ± 2.52 ^ab^	52.68 ± 2.66 ^b^	0.03
	Post	71.93 ± 6.40 ^a^	55.81 ± 2.74 ^ab^	51.80 ± 3.06 ^b^	0.009
Triglycerides (mg/dL)	Pre	99.13 ± 15.84 ^a^	93.33 ± 7.71 ^a^	128.6 ± 9.12 ^b^	0.008
	Post	133.3 ± 28.83 ^a^	101.5 ± 7.76 ^a^	148.1 ± 15.27 ^a^	0.05

BMI: Body Mass Index; NW: Normal Weight; OW: Over-Weight; OB: Obese; TSH: thyroid-stimulating hormone; fT3: free triiodothyronine; fT4: free thyroxine; γGT: gamma-glutamyl transferase; ALT: alanine aminotransferase; AST: aspartate aminotransferase; HDL Cholesterol: High-Density Lipoprotein Cholesterol. Data are expressed as means ± SEM. Data within the groups were analyzed using the Wilcoxon rank sum test (pre- and post-exercise intervention). The Kruskal Wallis test with Dunn’s multiple comparison tests was applied to verify differences among groups pre- and post-exercise intervention; different letters differ significantly (*p* < 0.05).

**Table 3:** Effects of Fitwalking on the markers of intestinal barrier integrity and fermentative dysbiosis according to BMI at the baseline and after exercise intervention.

		NW (n. 15)	OW (n. 24)	OB (n. 18)	*p*-Value
Serum zonulin (ng/mL)	Pre	40.91 ± 3.37 ^a^	50.60 ± 3.93 ^ab^	57.54 ± 4.74 ^b^*	0.01
	Post	38.97 ± 2.66 ^a^	46.93 ± 3.13 ^a^	47.02 ± 3.83 ^a^*	0.20
Fecal zonulin (ng/mL)	Pre	90.13 ± 10.69 ^a^	116.40 ± 16.29 ^a^**	120.60 ± 20.52 ^a^	0.53
	Post	105.70 ± 17.46 ^a^	150.3 ± 21.18 ^a^**	121.80 ± 22.53 ^a^	0.37
I-FABP (ng/mL)	Pre	4.08 ± 0.33 ^a^	3.69 ± 0.38 ^a^	4.36 ± 1.31 ^a^	0.25
	Post	4.26 ± 0.17 ^a^	3.44 ± 0.38 ^a^	3.93 ± 0.98 ^a^	0.06
Indican (mg/L)	Pre	42.95 ± 5.46 ^a^	61.22 ± 6.79 ^a^	85.22 ± 12.67 ^a^*	0.64
	Post	44.89 ± 6.45 ^a^	57.74 ± 6.69 ^a^	58.62 ± 8.61 ^a^*	0.40

BMI: Body Mass Index; NW: Normal Weight; OW: Overweight; OB: Obese. I-FABP: Intestinal Fatty Acid Binding Protein. Data are expressed as means ± SEM. Data within the groups were analyzed by the Wilcoxon rank sum test (pre- and post-exercise intervention: */** *p* < 0.05). The Kruskal Wallis test with Dunn’s multiple comparison tests was applied to verify differences among groups pre- and post-exercise intervention; different letters differ significantly (*p* < 0.05).

**Table 4:** Regression analysis between basal Global Physical Capacity Score (GPCS) levels and clinical and biochemical variables at the start of the study.

Parameters	β	Std. Error (β)	*p*-Value	95% CI
Body Mass Index	−0.067	0.032	0.043	−0.004–−0.129
Serum zonulin	−0.033	0.010	0.001	−0.014–−0.052

## Data Availability

Data are available upon reasonable request.

## Supplementary Materials

The following supporting information can be downloaded at: https://www.mdpi.com/article/10.3390/nu16234181/s1, Table S1: Anthropometric and bioimpedance characteristics of IBS patients stratified by BMI and sex at baseline (Pre) and after (Post) the exercise intervention.
