# Supplementary material for: Beyond Nutritional Treatment: Effects of Fitwalking on Physical Capacity and Intestinal Barrier Integrity in BMI-Stratified IBS Patients
Source: Nutrients. 2024 Dec 2;16(23):4181. doi: 10.3390/nu16234181 (PMC11644420; doi:10.3390/nu16234181)
Supplement: Supplementary file 1 [file nutrients-16-04181-s001.zip › nutrients-3340969-supplementary.pdf]

Table S1. Anthropometric and bioimpedance characteristics of IBS patients stratified by BMI and sex at baseline (Pre) and after (Post) the exercise intervention.

		NW (n. 15)		OW (n. 24)		OB (n. 18)	
**Sex (Male/Female)**		M	F	M	F	M	F
**N**		2	13	9	15	4	14
		Mean ±SEM	Mean ±SEM	Mean ±SEM	Mean ±SEM	Mean ±SEM	Mean ±SEM
**Waist Circumference**	Pre	90.00±1.00	76.69±1.34	98.87±1.04	86.93±1.33	117.2±5.99	104.5±1.80
	Post	88.75±1.75	75.95±1.45	97.21±1.40	85.37±1.42	115.9±6.19	103.2±1.82
**Hip Circumference**	Pre	95.50±0.50	97.73±1.48	102.6±1.61	103.8±1.81	110.0±7.38	117.8±2.83
	Post	95.50±2.00	97.59±1.57	101.4±1.80	102.1±1.64	109.8±7.53	115.9±2.79
**Waist/Hip Ratio**	Pre	0.94±0.01	0.77±0.02	0.96±0.01	0.83±0.01	1.06±0.02	0.81±0.06
	Post	0.92±0.00	0.78±0.02	0.95±0.01	0.83±0.01	1.06±0.02	0.89±0.02
**PhA (degrees)**	Pre	7.40±0.80	5.71±0.22	7.03±0.35	6.03±0.18	7.55±0.25	6.46±0.13
	Post	6.14±0.19	6.14±0.19	6.80±0.22	6.06±0.19	7.12±0.13	6.50±0.25
**BCM (kg)**	Pre	35.10±4.30	22.75±0.78	36.03±1.19	23.95±0.52	41.52±1.96	28.77±0.57
	Post	36.25±4.15	23.42±0.65	34.69±1.29	24.27±0.73	39.80±1.93	28.80±0.53
**FM (kg)**	Pre	14.45±0.85	20.80±3.24	22.50±2.90	23.59±1.44	37.30±7.88	36.41±2.54
	Post	13.55±2.15	16.67±0.86	22.59±1.92	22.85±1.19	37.32±8.93	38.17±2.59
**FFM (kg)**	Pre	58.30±4.10	43.78±1.21	61.90±1.70	44.62±0.92	68.62±3.88	51.51±1.00
	Post	58.70±3.60	43.12±0.75	60.35±1.93	45.09±1.04	67.57±3.81	51.74±0.96
**TBW (liters)**	Pre	42.45±2.95	32.09±1.02	44.93±1.40	32.41±0.74	50.07±2.93	37.73±0.78
	Post	42.35±2.25	31.37±0.56	40.07±1.45	32.86±0.81	49.37±2.87	37.83±0.76
**ECW (liters)**	Pre	16.95±0.05	15.20±0.67	18.75±0.91	14.83±0.51	19.87±1.53	16.53±0.45
	Post	16.35±0.35	14.17±0.37	18.67±0.72	15.01±0.49	20.25±1.42	16.65±0.65

IBS: Irritable Bowel Syndrome; BMI: Body Mass Index; M: Male; F: Female; N: Number of subjects; NW: Normal Weight; OW: Overweight; OB: Obese; PhA: Phase Angle; BCM: Body Cell Mass; FM: Fat Mass; FFM: Fat-Free Mass; TBW:Total Body Water; ECW: Extracellular Water.
